# Food-Sustainable Behaviors and Attitudes of Generation Z Consumers—Measurement and Analysis of Selected Behaviors

**DOI:** 10.3390/foods15081310

**Published:** 2026-04-10

**Authors:** Agata Balińska, Ewa Jaska, Agnieszka Werenowska

**Affiliations:** 1 Institute of Economics and Finance, Warsaw University of Life Sciences, Nowoursynowska St. 166, 02-787 Warsaw, Poland; ewa_jaska@sggw.edu.pl; 2 Management Institute, Warsaw University of Life Sciences, Nowoursynowska St. 166, 02-787 Warsaw, Poland; agnieszka_werenowska@sggw.edu.pl

**Keywords:** sustainable consumption, food, generation Z, mobile applications, community gardens

## Abstract

Food waste in households means that there is a need to recognize the possibilities of balancing activities in the field of obtaining and managing food products. Activities in this area may concern giving away surplus food to others, purchasing local and organic products, limiting shopping activity. Generation Z, which was included in this research, uses new media, including mobile applications, to a greater extent than other generations. The main objective of the research is to recognize and present the food-sustainable behaviors and attitudes of Generation Z consumers. The study used the analysis of source data, which was the basis for formulating four hypotheses. They were verified in empirical studies conducted using the CAWI method. The collected material was analyzed using, among others, the proprietary index of environmentally and socially sustainable behaviors (ESRBI), the Mann-Whitney test. The studies showed that respondents assessed their food behaviors as irresponsible, with women’s assessment being higher than men’s. A positive correlation was demonstrated between the use of food saving applications and the value of the ESRBI index and individual sustainable behaviors. Respondents positively assessed the initiatives of local authorities and housing cooperatives in the area of creating places for sharing food and organizing community gardens.

## 1. Introduction

Sustainable behavior, particularly in the case of food, can be analyzed from the perspective of various scientific theories. The present study considers the classic aspects of sustainable consumption: environmental, social, and economic. The concept of sustainable consumption was first presented at the Earth Summit in Rio de Janeiro in 1992. The Global Sustainable Development Goals were formulated at that time, and in 2012, the United Nations Conference on Sustainable Development, commonly known as “Rio+20”, was held. The result was a document that contained specific and practical measures to implement the idea of sustainable development. In 2015, the Agenda for Sustainable Development until 2030 was signed, defining a 15-year plan to achieve these goals [1]. Sustainable food consumption is of great importance in solving global environmental problems and promoting health and ethical behaviors. Recognizing the determinants of sustainable food choices, especially among younger generations, e.g., Generation Z, is necessary to develop solutions to prevent food waste in the future. This is a multifaceted issue and occurs at various stages throughout the food supply chain, from production to consumption. It is estimated that almost one third of the total mass of food and about one quarter of the total number of calories produced in the world is lost or wasted [2]. In Poland, the problem of food waste is becoming increasingly noticeable. Every year we lose about 4.8 million tons of food, 60% of which comes from households [3]. In addition to economic costs, food waste has serious environmental and social consequences, such as contributing to greenhouse gas emissions and climate deterioration. Avoiding food waste has become a subject of interest for many researchers in recent years. Among others, the problem of building awareness and creating pro-environmental behaviors of Generation Z has been engaged in order to reduce food waste. Studies on Generation Z clearly indicate that they are more motivated to protect the environment and better educated in the field of sustainable lifestyle than previous generations [4]. According to the study by Qi et al. [5] subjective norms, attitudes and perceived behavioral control positively influence generation Z intentions to avoid food waste. In turn, the results of the study conducted by Jakubowska et al. [6] show that attitudes and knowledge are significant determinants of sustainable food consumption among generation Z representatives, while subjective norms, perceived behavioral control, health awareness and trust do not significantly affect purchase intentions. The discrepancies in the results can probably be explained by regional conditions, because the first study was conducted in China and the second in Poland. That is why comparative analyses are so important. Another example can be the study of the organic food market in Germany and Poland. The countries are the leading producers of this type of food in Western and Middle Eastern Europe, respectively. It has been shown that Polish and German generation Z consumers present pro-ecological attitudes, but the organic food market is developing at a much faster pace in Germany [7]. For young consumers belonging to Generation Z, an important criterion for choosing products and services is not only the impact on the environment, but also the impact of these products on their health and general well-being. This was also confirmed in their research by Kymäläinen, et al. [8].

In-depth analyses of consumer attitudes are also becoming important to determine trends in the development of the food market, and so, for example, Chen et al. [9] present a comparative analysis of Generation Z and Y, indicating a number of common features in the choice of local food, including health awareness, subjective norms, perceived behavioral control and attitude. However, there is a significant discrepancy in the factors motivating purchases. Convenience is the main determinant of Generation Z when choosing local food, and price is a decisive factor in the decision-making process of Generation Y.

In such conditions, the importance of educational and marketing campaigns that increase consumer knowledge and shape a positive attitude towards sustainable consumption increases. In Canada, mobile technology and applications have been identified as a potential solution for redistributing food (before it becomes waste), raising awareness about food waste, and supporting behaviors that reduce food waste [10]. As researchers rightly point out, placing an exclusive emphasis on mobile applications can result in the burden of food waste falling on those at the end of the food supply chain. Reducing food waste must be part of an integrated plan of action at the government, industry, and individual levels. Mobile applications alone will not eliminate household food waste without the right public awareness, resources, or infrastructure. Generation Z, including people born after 1995, is characterized by high environmental awareness and a tendency to use modern technologies in everyday life. Currently, there are several mobile applications aimed at minimizing food waste by facilitating the sharing of surplus food, e.g., Olio, Too Good To Go, Karma, NoWaste, Nosh, FoodCloud, Kitche or Foodsi.

Hong et al. [11] rightly noted that there is no single general definition of mobile applications used to reduce food waste and, based on the analysis of the literature on the subject, they cite such terms as: mobile applications for food sharing, applications for managing food waste, digital applications for reducing food waste, applications for food redistribution or applications for managing food in households. The authors proposed the following definition of mobile applications dedicated to reducing food waste (FWMA): they connect consumers and food companies, thanks to which the latter can offer consumers food products at reduced prices in order to reduce food waste. These transactions have a positive social, environmental and economic dimension, as the food company receives remuneration, the consumer pays a reduced price for the product, food with a short shelf life is consumed and its waste is reduced.

An interesting solution is described by Mohod et al. [12] who present a mobile application enabling the sharing of home-cooked meals in a corporate environment. Suppliers can publish their meal boxes with detailed information such as location, type of food and availability status, and consumers can search and select meal boxes based on their preferences. On the other hand, Xi-Yu et al. [13] assessed the behavioral intentions of Taiwanese Generation Z consumers and found that Generation Z’s engagement with food processing videos on streaming platforms is positively correlated with their subsequent purchasing behavior. The results of studies conducted by Stanes et al. [14] and Fisher et al. [15], in which representatives of Generation Z indicated that it is relatively easy to avoid food waste, are also encouraging. It is particularly important to state that their attitudes towards food are determined by the customs prevailing in their families. Sustainable food behaviours of consumers concern not only the handling of products that they purchase on the market, but also those that they produce themselves. An interesting trend in recent years is urban agriculture, including community gardens, i.e., initiatives leading to the arrangement of places for growing vegetables and fruit. Community gardens (communal, municipal) are an element of the so-called urban agriculture, i.e., an approach in which cities act not only as food consumers, but also as producers [16]. Jac Smit, considered the creator of urban agriculture, claims that the development of this concept will contribute to the introduction of a more sustainable economy and may in the future result in a reduction in the scale of hunger and poverty in urban areas [16,17]. According to Butterfield’s [18] research, the main reasons for establishing community gardens are both economic and non-economic, which fits into the broader context of the idea of sustainable development.

The main objective of the article is to identify and present the behaviors and attitudes of Generation Z consumers in terms of food sustainability. The main objective formulated in this way allowed for the identification of the following specific objectives:

**C1.**  *Determining the relationship between the perceived importance of sustainable consumption and the declared behaviors in this area.*

**C2.**  *Verifying the difference in sustainable food consumption between women and men.*

**C3.**  *Determining the role of food saving applications in creating positive behaviors in the area of sustainable food consumption*

**C4.**  *Identifying the opinions of Generation Z consumers on food-sharing units and community gardens established by local authorities, cooperatives and housing communities.*

**Figure 1:**
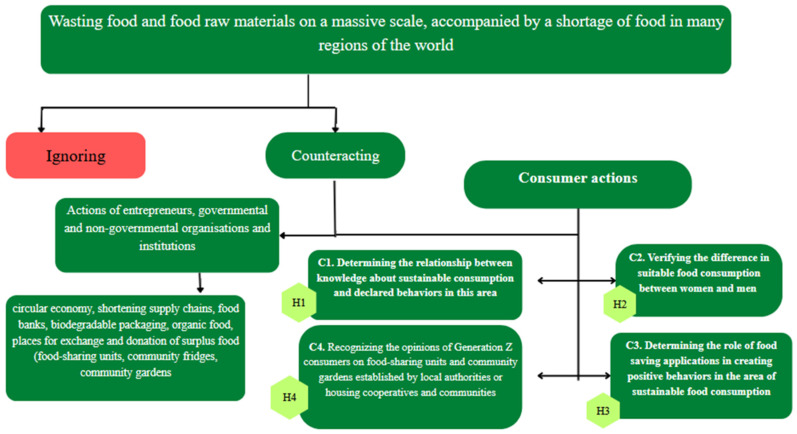
Conceptual model. Source: own research.

The research hypotheses are related to the above-mentioned objectives, as illustrated in the conceptual model (Figure 1).

The structure of the article is divided into 4 sections. In the first, “conceptual framework of research”, an analysis of the literature on the subject was carried out and 4 research hypotheses were formulated. The second part is a presentation of the methodology of own empirical research, including a measure that allows for describing sustainable behaviors by a numerical value. The third is a presentation and discussion of the results of the empirical research. The last part of the article is a discussion and conclusions, in which the advantages of the presented research, their limitations, practical implications are indicated and the direction of further research in this area is determined.

## 2. Conceptual Framework of the Research

With the increasing pace of environmental degradation, climate change and resulting crises, the need to change consumption behaviors to more sustainable ones is becoming increasingly important [19]. The relationship between environmental concern and sustainable consumption behavior requires continuous and interdisciplinary analyses [20]. The study by Panopoulos et al. [21] shows that environmental concern, eco-labels, influencers and user-generated content influence the intention to purchase green among Generation Z. Similarly, Saari et al. [22] draw attention to the need for research on the relationship between environmental knowledge, environmental concern and behavioral intentions and behaviors related to sustainable consumption in Europe. Sustainability content on social media is considered as a stimulus that influences the sustainable behavior of Generation Z. Confetto, et al. [23] conducted a study among Italian representatives of this generation and found that exposure to sustainable development content on social media affects their responsible consumption behavior. Similarly, households equipped with greater knowledge of energy saving techniques show a greater propensity to adopt energy-saving behaviors [24]. Consumer knowledge plays a key role in shaping buyer behavior, especially in the context of a sustainable lifestyle and the problem of food waste. Recognition of the determinants of sustainable consumption attitudes among young people was the subject of an analysis conducted by Hong, et al. [25]. Similarly, Naz Onel Avinandan Mukherjee [26] indicates that concern for the environment and knowledge positively influenced the adoption of responsible consumption practices. In turn, research conducted by Saulais and Espougne [27] indicates the need to improve awareness of the impact of food production on the environment, thus emphasizing the importance of effective information and educational strategies in shaping sustainable eating habits. Consumers declare concern about environmental issues, but this is not reflected in the presented lifestyle. Therefore, consumer education becomes a key element supporting a sustainable lifestyle and thereupon the following hypothesis was formulated:

**H1.**  *There is a discrepancy between the perceived importance of sustainable consumption and the declared behaviors in this area.*

Sustainable consumer behavior is also analyzed according to gender and the study was conducted, among others, in the United Arab Emirates. The study showed that there are differences between the genders in terms of efficient use of resources, ecological initiatives and minimizing waste. Women’s purchasing decisions were significantly more shaped by sustainable consumption patterns compared to men. Studies indicate that women are more likely to make purchasing decisions in line with the principles of sustainable development and are characterized by greater concern for the environment [28]. Bulut et al.’s [29] and Brough et al.’s [30] research also confirmed the relationship between gender and sustainable consumption behaviors, e.g., women were more likely to reuse products. Their sustainable attitudes are more visible in the household, e.g., saving water and energy. In turn, Zelezny et al. [31] state that men avoid sustainable products and behaviors that are more strongly associated with women. Dahl et al. [32] also indicate differences in sustainable consumption and state that women are more likely to buy organic food and sustainably produced clothing. The results suggest that gender and generation of consumers may differentiate sustainable consumption behaviors, which is also confirmed in the research of Bloodhart and Swim [33]. Therefore, another hypothesis was formulated:

**H2.**  *Women significantly higher than men rate their own behavior in the area of sustainable consumption*.

Sharing food is not a new phenomenon. This phenomenon has been known for a long time, particularly among residents of villages and small towns, but nowadays, especially in large cities, it takes a different form, namely applications and online platforms [34,35,36]. The issue of mobile applications developed to prevent food waste was analyzed, among others, by Doğan et al. [37]. Their results indicate that people who care about sustainable development, care about not wasting food and shape their sustainable shopping behavior through mobile applications. In turn, Ng et al. [38] present a comprehensive model that identifies the influence of internal and external factors on the intention to constantly use food applications. Although the results of their study show that saving time is the most important factor influencing the perception of the application by customers, also important are the different food options and the usability of the application, which affects the continuous use of these services and the shaping of sustainable behavior. An interesting experimental study using a fictitious mobile application called “Boxy Food” was conducted by Sestino et al. [39]. The participants of the study were a group of 408 randomly selected people from different countries. The results of this analysis indicate that focusing on communication related to sustainable development in campaigns has a positive impact on consumers’ intentions to use these applications and protect the environment. Poles have been using the TooGoodToGo and Foodsi applications for several years. The TooGoodToGo application (current data on the number of users is not publicly available but in 2015 175 thousand business partners and about 100 million users were registered worldwide [40]) is based on connecting users with local restaurants, bakeries, grocery stores and other catering outlets that offer unsold food at the end of the day. The application is a very good solution for those who value a sustainable lifestyle. In turn, Foodsi, a Polish application launched in 2019, has established cooperation with over 5000 business partners by December 2023 and has gathered over 1.5 million users. Thanks to these efforts, it was possible to save over 2 million food packages [41]. The application works on a similar principle to TooGoodToGo, but focuses on the local market, offering support to both users and the catering industry in Poland. The aforementioned applications operate on similar principles and are the most popular applications of this type in Poland. Therefore, the following hypothesis was formulated:

**H3.**  *There is a positive relationship between the use of food saving applications and positive behaviors in the area of sustainable consumption*.

The development of social initiatives, in the form of food-sharing units (“social fridges”) or social gardens, makes these initiatives increasingly the subject of interest of scientists. Scientific research on social gardens in cities indicates that the benefits of developing this concept are multifold. Gardens provide fresh products of known origin and acceptable cost [42,43,44,45]. Lee and Matarrita-Cascante’s [46] research shows that community gardens in Austin, Texas produce about 100,000 pounds of fresh, local, and organic produce annually, and food production remains the most important motivator, apart from recreation and education. Schoen et al.’s [47] research also points that community gardens provide specific economic benefits (return on investment of £3 for every £1 invested). Furness and Gallaher [48] present interesting results of a qualitative study. It turned out that consumers who were forced to make selections of food products for economic reasons usually gave up buying fruit and vegetables, indicating that they were expensive. Therefore, according to the authors, community gardens can offer fruit and vegetables, and an additional advantage is the proximity to the place of residence of consumers. They also found that consumers prefer products from the common garden to those bought in the store, and “freshness” and the certainty that they do not contain pesticides were the most common reasons for such declarations.

Community gardens also mean greening of the space, which is beneficial for mental health. This applies especially to tall plantings, i.e., shrubs and fruit trees. Spending time in such spaces increases resistance to stress [49,50].

An important function of community gardens is education in the field of the principles of horticultural production, the functioning of ecosystems, the use of vegetables and fruits in the preparation of specific dishes, which help shape healthy eating habits, especially amongst young people. This aspect is emphasized by, among others, Grier et al. [51] and Ornelas et al. [44]. The research of Grier et al. [51] shows that the educational function is particularly important for residents of apartment blocks who do not have their own gardens. It is combined with the recreational function of gardens. Rest and relaxation in the fresh air and manual work reduce stress. The therapeutic function of gardens is also indicated by Lin et al. [43], Schoen et al. [47].

The integration of the local community through voluntary involvement in a common project and spending time together is very important. Gardens are often a space for building intercultural [52] and intergenerational [53] relations. Building a spatial community involves not only joint cultivation, but also joint cooking and joint meals [49,54]. Liamputtong and Sanchez [55] point out that gardens provide an opportunity to assimilate for people new to a given area, which is very important in times of intensive migration, especially of young people.

Many researchers also point out that such initiatives cannot be implemented only at grassroots without the involvement of external entities, e.g., (non-profit organizations, local authorities), which is related to both providing space for a garden and specific know-how in the field of the principles of growing vegetables and fruit. In turn, Drake and Lawson [56] emphasize that although each community garden is shaped by the local context, all of them have certain features that distinguish them from commercial agriculture and home gardening. People work in them on a voluntary basis, but they need the support of people with substantive knowledge. The studies by Menconi et al. [57], Furness and Gallaher [48] show that the functioning of gardens is better the greater the support from external entities. The inclusion of Generation Z in the issue of community gardens results from the fact that it is important to connect people from different generations [53].

The need for simultaneous implementation of various food sharing initiatives was indicated in their studies by, among others, Davies and Evans [58], Tatebayashi et al. [59], Zhao et al. [60]. Such a way are food-sharing units, i.e., places equipped with refrigerators and shelves, where everyone can anonymously leave surpluses of full-value food, and those in need can take it free of charge. Consumer involvement in food redistribution is very important, because in the EU almost 59 million tonnes of food are thrown away every year, which means 131 kg per person, and more than half comes from households [61]. Food-sharing units are good places to donate even small amounts of food or ready meals. They are most often located on the premises of public facilities, including city and commune offices, etc. Despite the relatively rich literature on the analysed initiatives, studying the attitudes of Generation Z towards this type of activity is a clear research gap. The scientific discussion indicated above prompted the authors to adopt the following research hypothesis:

**H4.**  *Respondents positively assess the activities of local government and housing cooperatives and communities that improve access to free raw materials and food products*.

## 3. Materials and Methods

In addition to analyzing scientific publications, the article draws on the results of our own survey bands. The empirical research used the CAWI technique. The survey questionnaire included sustainable food behaviors analyzed by the various researchers cited in this article. Both the list of these behaviors and the questionnaire design were tested in pilot studies conducted in 2021. Selected results from the pilot studies were subject to scientific discussion through presentations at scientific conferences and a publication [61,62]. The experience gained at this stage allowed us to refine the research tool used in the main research. The research was carried out in 2023.

The questionnaire was prepared on the webankieta platform. The questionnaire included questions with a 5-point scale, which allowed for determining the importance of specific food behaviors and the self-assessment of these behaviors by respondents. Questions with a 5-point scale were also used to assess the activities of local government, cooperatives and housing communities.

Therefore, the study used an ordinal scale. There is an ongoing debate in the scientific literature [63,64] about whether the same statistical measures can be applied to ordinal scales as to quantitative scales (mean, standard deviation), or whether one should limit oneself to the median and quartiles, as well as nonparametric tests (including Mann-Whitney and Kruskal-Wallis). Due to numerous reservations expressed by researchers [65,66] and our own research experience, this study adopted a classical approach, recommending the use of the median, quartiles, and nonparametric tests.

Alternative questions (to verify the use of food rescue applications by respondents, i.e., TooGoodToGo and Foodsi) and single-choice questions (to obtain information on the socio-demographic profile of respondents) were also used.

The study was fully anonymous, respondents were informed about the purpose of the study and were not asked to provide sensitive data and could withdraw from the study at any stage. Taking into account the communication style of Generation Z, social media profiles and platforms [67,68,69,70,71,72] were used to reach the widest possible group of respondents. Therefore, convenience sampling [73,74,75,76,77], popular in social research, was used, as well as the snowball sampling method [78,79,80]. Respondents were asked to share a link or QR code with their friends, which allowed for greater sample diversity. Each respondent was allowed to complete the survey only once. The collected material was subjected to quantitative and qualitative analysis.

While analyzing the possibilities of measuring sustainable behavior, the authors also sought new, not commonly used measurement methods.

The analysis of the research results used the Environmentally and Socially Responsible Behavior Index (ESRBI) calculated according to the formula [81]:

$$ESRBI=\sum_{i-1}^{N}W_{i}\cdot C_{i}$$

where:

ESRBI—result of environmentally and socially responsible behaviors

i—consecutive number of the studied behavior,

N—number of behaviors determined in the analysis,

W_i_—coefficient of significance of the weight of the i-th behavior,

C_i_—self-assessment of the i-th behavior by the respondent

The value of the index is interpreted as follows: 0–40.0—very irresponsible behaviors, 40.1–60.0 irresponsible; 60.1–75.0 indifferent, 75.1–90.0—responsible; 90.1–100 very responsible.

The ESRBI was constructed using the CSI (Customer Satisfaction Index) methodology, which is frequently used in scientific research [82,83,84,85,86,87]. It was also used by the authors of this study [88,89]. The obtained result can be analyzed in absolute values. However, this would pose a significant difficulty in comparing results, as the CSI does not have a mandatory scale range (the only requirement is odd scale degrees). This problem was pointed out in their studies by, among others, Yadav et al. [90]. Analyzing studies conducted using various scales, these authors indicated that a percentage scale is a good solution [90]. The interpretation of the CSI result is as follows: 0–40% very bad (extremely dissatisfied); 40–60% bad (dissatisfied); 60–75%—average (there are some problems with user satisfaction); 75–90%—good (there are minor problems with user satisfaction); 90–100%—very good (highly satisfied) [82,91]. Therefore, as in the case of the CSI, a percentage scale was used, with analogous ranges. The authors also have doubts whether the adopted thresholds are adequate for measuring sustainable behavior. Therefore, they have submitted this method of measurement to scientific discussion in peer-reviewed scientific publications [81,92] and presentations at scientific conferences. This publication is also an invitation to further discussion on this topic.

In addition to the ESRBI index, the analysis used the median, quartiles, Mann-Whitney test, Kruskal-Wallis test, ANOVA analysis of variance, and Levene’s test.

## 4. Results

844 adult representatives of Generation Z participated in the study. After verification of the correctness and completeness of the questionnaires, 832 correctly completed questionnaires were qualified for analysis. The reliability of the questionnaire was checked using the Cronbach’s Alpha test, the value of 0.808 indicates that it is reliable. The group was dominated by women (61.1%), men constituted 36.5% and the remaining 2.4% did not indicate gender. Other researchers, including Mulder, de Bruijne [93], Morgan [94], Slauson-Blevins and Johnson [95] Smith [96], Keusch [97] draw attention to the common dominance of women in survey studies.

To compare men and women, Levene’s test for homogeneity of variance was performed. A statistical significance of *p* = 0.878 indicates that the result is statistically insignificant, meaning the variances are homogeneous.

Every third respondent (33.3%) lived in cities with more than 500,000 inhabitants, 33.1% in the countryside; 22.8% in cities with up to 50,000 inhabitants, and the remaining 10.9% in cities with 50 to 500,000 inhabitants. More than half of the respondents (56%) lived in a single-family home, 38.2% in a block of flats, and only 5.8% indicated a boarding school or a student dormitory. The largest share was made up of respondents declaring living in 4-person households (45.9%), then 5-person (20%), 3-person (17.9%). Only 1.6% indicated that they lived alone, and 4.3% in a two-person household. The remaining respondents indicated at least 6-person households.

The age of the respondents was related to their main source of income, namely almost half of them lived off payments from parents and other family members, which is natural in Poland because young people are usually supported by their parents during the period of continuing education. Almost every fourth respondent (23.3%) lived off permanent employment, and 22.2% off contract work. Only for 5.1% of respondents, the main source of income was academic or social scholarships, and for 1.4% social benefits (annuities). The largest share was made up of respondents who spent up to PLN 1500 on their living (46.7%). Every third (33.8%) indicated an amount of PLN 1500–3000, and 19.6% above PLN 3000.

Every fourth respondent (24.8%) declared using the TooGoodToGo application, significantly fewer, only 6.9%, declared using the Foodsi application.

In accordance with the adopted hypothesis H1, the occurrence of discrepancies between the perceived importance of sustainable behaviors and the declared behaviors was positively verified. For this purpose, the ESRBI index was used (Table 1), the formula of which is provided and described in the Section 3.

The ESRBI index value of 55.9 (Table 1), in accordance with the adopted interpretation, indicates that the respondents’ behaviors are irresponsible. It should be noted that in relation to all the analyzed behaviors, their importance assessments are on average higher than the respondents’ assessment of the same behaviors in themselves, with the greatest difference being visible in the behavior of giving away and/or obtaining items to and from the food-sharing units (Table 1).

Statistically significant differences between the perceived importance of individual behaviors and their practice were also confirmed using the Anova test. In each case, the level of statistical significance indicated (*p* < 0.05) that the differences were statistically significant. Therefore, the obtained measurement results are compatible.

In accordance with the adopted hypothesis H2, it was verified whether women assess their own behaviors in the area of sustainable consumption significantly higher than men. The ESRBI index value for women was 58.7, and for men 51.8. Verification with the Mann-Whitney Z test showed that women assess their own behaviors significantly higher than men, which is illustrated in Table 2.

Only in the case of purchasing items with the Fairtrade mark and limiting purchasing activity was there no statistically significant difference between women and men (Table 2). It should be emphasized that the Fairtrade mark is found primarily on chocolate packaging (in Poland in 2023, 47.5 million chocolate bars with this marking were sold), the share of which in products with this mark is as much as 89% (coffee is in second place 9% and other products constitute 2%) [98].

The conducted study also attempted to determine the relationship between the use of food saving applications and positive behaviors in the area of sustainable consumption (H3).

The value of the ESRBI index for consumers using the TooGoodToGo application was 62.8, and for those not using this application 55.7. In the case of respondents using the Foodsi application, it was 64.04, and for those not using this application, it was 55.4. An analysis using the Mann Whitney U test was also conducted, which showed that respondents using the TooGoodToGo application rated their behaviors significantly higher than those not using this application in terms of: using food-sharing units (*p* = 0.00578), shopping in “zero waste” departments (*p* = 0.0001), reducing meat consumption (*p* < 0.00001), buying food products in recycled packaging (*p* = 0.0056), buying organic food (*p* = 0.00168), using reusable bags for fruit and vegetables (*p* = 0.00034). However, they rated their behaviors in terms of reducing shopping activity lower (*p* = 0.0001). There was no statistically significant difference in the case of purchases with the Fairtrade label (*p* = 0.12356) and purchases of products from domestic producers (*p* = 0.28914). Also, respondents using the Foodsi application rated their behavior in terms of using food-sharing units higher than those not using them (*p* = 0.0083). However, there was no statistically significant difference in relation to purchases in “zero waste” sections (*p* = 0.2113), reducing shopping activity (*p* = 0.29372), reducing meat consumption (*p* = 0.33204), purchasing products in recycled packaging (*p* = 0.96012), purchasing organic food (*p* = 0.1556), using reusable bags for fruit and vegetables (*p* = 0.92034), purchasing food with the Fairtrade label (*p* = 0.238) and purchasing products from domestic producers (*p* = 0.74896). Thus, H3 was positively verified: There is a positive relationship between the use of food saving applications and positive behaviors in the area of sustainable consumption.

In accordance with the adopted H4, it was verified whether respondents positively assess the activities of local government and housing cooperatives and communities improving access to free raw materials and food products (Table 3).

Respondents living in rural areas may not have experience in using the initiatives listed in Table 3. They are typical for cities, especially large cities (e.g., in Warsaw there are 47 food-sharing units [99] and 18 community gardens [100]. However, this does not mean that city residents have knowledge about them. Furness and Gallaher [48] also point this out. Respondents who have contact with such initiatives do not agree in their assessments, but the percentages of people who assess them positively (total options “rather good” and “very good”) and negatively (options “definitely low” and “rather low”) are higher in the latter case, with the difference being the largest in the assessment of the establishment of community gardens by cooperatives and housing communities (35 p.p.), and the smallest in the case of planting fruit trees and shrubs instead of ornamental ones by the local authorities (1.6 p.p.). Hypothesis H4 was verified positively.

The low score may be due to the lack of involvement of respondents in such initiatives, an example of which is donating food to a food-sharing units, which was rated 1.8 points by respondents (Table 1) and was the lowest rated behavior.

## 5. Discussion and Conclusions

The currently observed high level of food waste in households means that there is a need to recognize the possibilities of limiting this phenomenon. In order to develop specific proposals to counteract the problem, it is first necessary to characterize the currently presented attitudes in different age groups, because they are diverse. The authors recognized the behavior of representatives of Generation Z, who notice environmental problems, including sustainable consumption, and put forward the thesis (H1) that there is a discrepancy between the perceived importance of sustainable consumption and the declared behaviors in this area.

When analyzing the results of other researchers, it should be emphasized that perceived importance is not the only determinant and its impact should be considered together with other conditions. Bauerne Gáthy et al. [101] in their research among students of the University of Debrecen noticed that they perceive themselves as health-conscious food consumers and tend to reject the environmental aspects of food consumption. In addition, their decisions regarding food consumption are dominated by self-interest over socially responsible behavior. The influence of knowledge, beliefs and experiences was analyzed, among others. amongst urban youth in the USA on their intentions to purchase Fair Trade products [102]. It turned out that consumers’ knowledge about Fair Trade FT has a positive impact on their purchase intentions, although such attitudes are reinforced by beliefs and previous behaviors. Filip et al. [103] identified the main factors influencing the intention to purchase eco-products among Generation Z representatives. According to the study, such determinants are: perceived quality of environmentally friendly products, consumer knowledge about eco-products and consumer trust. In turn, Robichaud and Yu [104] analyzed the intention to purchase Fair Trade coffee by Generation Z representatives and it turned out that in this case there is a significant impact of knowledge about Fair Trade on the interest in these products. Alves et al. [105] identified a positive attitude of students in Portugal towards behaviors in the area of the circular economy (including reducing food waste, recycling practices or buying energy-saving products). It has been confirmed that knowledge about the circular economy has a positive impact on shaping sustainable consumption among Generation Z representatives. The research conducted by Cebrián et al. [106] shows that students, when developing a list of policy recommendations in the area of 3R activities (reduce, recycle, reuse), indicate the need to limit the use and production of plastic packaging. This corresponds to the highly perceived importance of buying products in biodegradable packaging and using reusable fruit and vegetable bags confirmed in our research (Table 1).

The research assumption that women significantly higher than men assess their own behavior in the area of sustainable consumption (H2) was positively verified. Similarly, Le Hai and Larinow [107] indicate that women achieved significantly higher environmental identity scores than men. In turn, Galbreath and Tisch [108] analyzed the attitudes of people holding managerial positions and found that women in managerial positions are more sensitive to expectations regarding sustainable behavior—than their male counterparts.

Generation Z is distinguished by high environmental awareness and the use of modern technologies. They very often reach for mobile applications that help them reduce food waste and plan purchases [109]. The authors also hypothesized that there is a positive relationship between the use of food saving applications and positive behaviors in the area of sustainable consumption (H3). The results of the research conducted by Haas et al. [110] also confirm that mobile applications are perceived as promising tools for changing consumer behavior in order to ensure more sustainable food consumption. Searching for appropriate packages in applications is, on the one hand, a kind of fun, but reserving them and buying them is a reward, which, according to the approach proposed by Zhao et al. [111] has a significant impact on generating and maintaining sustainable behaviors. Despite the possibility of obtaining information through various sources, there is still a need to raise the environmental awareness of society, which is confirmed by Cebrián et al. [106]. Their research shows that this was the most important recommendation regarding educational and information policy reported by the study participants.

In accordance with the adopted H4, it was confirmed that respondents positively assess the actions of local government and housing cooperatives and communities that improve access to free raw materials and food products. For Generation Z, community gardens are part of the justice policy regarding food transformation, with particular emphasis on issues such as: availability, climate change, access to climate-friendly food, changes in food production, as well as cultural conflicts related to changing diets [112]. Spierings et al. [113] indicate similar regularities, claiming that community gardens, apart from the process of social inclusion, also lead to social exclusion. In Central and Eastern European countries, such as Poland, this is a new perspective on the manifestations of urban agriculture, unknown to previous generations. For millions of Poles raised in the times of real socialism (generation BB and X), workers’ allotments in cities provided primarily the opportunity to stock up on fruit and vegetables, which were too scarce on the market. To activate generation Z, new narratives about community gardens that correspond to their perception of the world are needed. The research by Lin et al. [43] shows that the functioning of gardens also requires additional programs dedicated especially to people with lower incomes. In the authors’ opinion, the entities that can achieve success in this respect in Poland are local authorities and housing communities and cooperatives, whose initiatives in the presented study were assessed positively by respondents. Reference should also be made to Kamiyama’s research [114], which shows that food sharing helps to create a sustainable and resilient food system, but is correlated with the culture of a given society and is exposed to imbalances resulting from, for example, health threats, as noted during the COVID-19 pandemic.

## 6. Implications, Limitations and Future Directions of Research

The presented study makes a significant contribution to the literature on sustainable consumption by providing information on the attitudes and behaviors of Generation Z consumers. From a practical point of view, the results of the study may be useful for many groups of recipients, including: food producers and sellers, representatives of environmental policy and broadly understood business. The results of the study indicate the need for continuous educational and promotional activities in the field of sustainable consumption.

Despite its cognitive value, the study has limitations that should be taken into account:

1. The study covered only a limited group of respondents from Generation Z, which makes it impossible to generalize the results to the entire population.
2. The results may depend on specific social and economic conditions, which limits their universality.
3. It cannot be unequivocally stated that a responsible approach to consumption prompted respondents to download the application or that having the application encourages them to behave responsibly. It should be emphasized that the study did not look for a cause-effect relationship.
4. The study presents a current picture of behaviors and attitudes, but does not take into account their evolution over time, which would allow for the depiction of observed trends in sustainable consumption.

In future studies, it would be advisable to extend the analysis with qualitative studies, which could deepen the understanding of the motivations and barriers related to making sustainable food choices among young consumers. Quantitative studies conducted in other European Union countries could be interesting.

## Figures and Tables

**Table 1:** Importance and assessment of behaviors in the field of sustainable consumption (on a scale of 1–5) and the ESRBI value.

	Specification	Perceived Importance of Behavior (M)	Weights	Behavior Score (M)	Weighted Result
1	reducing meat consumption	2.8	7.7	2.5	0.2
2	donating and/or obtaining products from food-sharing units	3.4	9.4	1.8	0.2
3	buying food in biodegradable packaging	4.2	11.6	3.3	0.4
4	purchasing organic food	3.2	9.0	2.7	0.2
5	shopping for food in “zero waste” sections	3.6	10.1	2.6	0.3
6	using reusable bags for fruit/vegetables	4.4	12.1	3.2	0.4
7	purchasing products with the FAIRTRADE mark	3.3	9.2	2.4	0.2
8	using applications that allow for “saving” food	4.1	11.4	2.8	0.3
9	purchasing food products from domestic producers	3.6	10.1	3.4	0.3
10	reducing purchasing activity of food products	3.4	9.4	3.1	0.3
	Results:	35.9	100		2.8 (55.9%)

Source: own research.

**Table 2:** Differences in the assessment of the behaviors of the surveyed women and men.

	Specification	Average Rank		Z	*p*
		Women *	Men **		
1	reducing meat consumption	2.868	1.828	11.335	0.000000
2	donating and/or obtaining products from food-sharing units	1.878	1.564	4.926	0.000001
3	buying food in biodegradable packaging	3.368	3.081	4.088	0.000044
4	purchasing organic food	2.866	2.500	4.912	0.000001
5	shopping for food in “zero waste” sections	2.752	2.385	4.797	0.000002
6	using reusable bags for fruit/vegetables	3.384	2.916	4.962	0.000001
7	purchasing products with the FAIRTRADE mark	2.440	2.311	1.795	0.072716
8	using applications that allow for “saving” food	2.996	2.382	6.216	0.000000
9	purchasing food products from domestic producers	3.355	3.345	−0.284	0.776598
10	reducing purchasing activity of food products	3.200	3.020	2.286	0.022234

* N weighted = 516; ** N weighted 296; Z = test value, *p*—level of statistical significance. Source: own research.

**Table 3:** Assessment of the activities of city and commune authorities and housing communities and cooperatives in pro-food areas.

		Assessment Level					
		I Don’t Know If It Is Implemented/I Have No Opinion	Definitely Low (1)	Rather Low (2)	Average (3)	Rather Good (4)	Very Good (5)
City and commune authorities	Establishing community gardens *	27.6	12.8	13.5	19.8	18.9	7.4
	Planting fruit trees and shrubs instead of ornamental ones *	19.0	14.2	13.6	21.6	20.4	11.2
	Organizing food-sharing units *	19.9	13.1	12.8	24.9	19.9	9.3
Cooperatives, housing and neighborhood communities	Establishing community gardens *	38.1	14.8	11.6	17.7	11.8	6.1
	Planting fruit trees and shrubs instead of ornamental ones *	34.2	13.2	12.9	18.6	14.3	6.7
	Organizing food-sharing units *	36.2	12.8	12.8	17.8	14.2	6.2

Source: own research; * Q_1_ = 2, Me = 3, Q_3_ = 4.

## Data Availability

The data presented in this study are openly available in RepOD (Repository for Open Data): https://repod.icm.edu.pl/dataset.xhtml?token=b69aebba-a12e-41f7-9164-d366abb48df9 (accessed on 26 May 2025).
